# Effect of intramolecular disulfide bond of bovine lactoferricin on its molecular structure and antibacterial activity against *Trueperella pyogenes* separated from cow milk with mastitis

**DOI:** 10.1186/s12917-020-02620-z

**Published:** 2020-10-23

**Authors:** Jie Pei, Lin Xiong, Min Chu, Xian Guo, Ping Yan

**Affiliations:** 1 Lanzhou Institute of Husbandry and Pharmaceutical Sciences, Chinese Academy of Agricultural Sciences, 730050 Lanzhou, China; 2 Key Laboratory for Yak Genetics, Breeding, and Reproduction Engineering of Gansu Province, 730050 Lanzhou, China

**Keywords:** Lactoferricin, Synthetic peptide, Antibacterial activity, *Trueperella pyogenes*, Mastitis

## Abstract

**Background:**

Lactoferricin (Lfcin) is an antimicrobial activity center of lactoferrin, produced by hydrolysis from the N-terminal of lactoferrin. It was hypothesized that the intramolecular disulfide bond in Lfcin could affect its antibacterial function through influencing its molecular structure. To prove this hypothesis, bovine Lfcin (bLfcin) and its two derivatives, bLfcin with an intramolecular disulfate bond (bLfcin DB) and bLfcin with a mutation C36G (bLfcin C36G), were synthesized, purified, and identified. The circular dichroism spectra of the peptides were detected in solutions with different ionic and hydrophobic strength. The antibacterial activity of the peptides against *Trueperella pyogenes*, separated from cow milk with mastitis, were determined.

**Results:**

The secondary structure of bLfcin DB showed more β-turn and less random coil than the other peptides in H_2_O, similar ratios of secondary structures with bLfcin and bLfcin C36G under ionic conditions, and close percentages of secondary structure with bLfcin under hydrophobic conditions. The synthetic peptides exhibited strong antimicrobial activity against *T. pyogenes* isolates, *T. pyogenes* ATCC 19,411, and *E. coli* ATCC 25,922. The antimicrobial activities of the three peptides were greater against *T. pyogenes* than against *E. coli*, and bLfcin DB exhibited higher antibacterial activity compared with its derivatives.

**Conclusions:**

The intramolecular disulfide bond could change the molecular structure of bLfcin under alternative ionic strengths and hydrophobic effects, and the formation of the disulfide bond is beneficial to executing the antibacterial function of bLfcin.

## Background

The resistance of pathogenic bacteria to conventional antimicrobial agents has become an increasingly serious threat to human public health [1, 2]. This situation is largely on account of antimicrobial agents overuse and excessive medical treatment [3, 4]. Antimicrobial peptides (AMPs) are an innate defense against natural microbial attacks, produced by immune system of organisms [5]. AMPs have multiple biological functions, including antibacterial, antiviral, anti-parasitic, anti-fungal. Moreover, the multi-functional mechanisms of AMPs reduce the potential to develop resistance of bacteria [6]. Based on this regard, peptides from host defense can be considered as molecular templates to design antibacterial agents to circumvent the increasing resistance of some pathogens.

Lactoferricin (Lfcin), a peptide hydrolyzed by gastric pepsin cleavage from the N-terminal region of lactoferrin [7], demonstrates much greater antimicrobial activity than lactoferrin [8–10]. The previous research showed that bovine Lfcin (bLfcin) has the strongest antibacterial function among many mammalian Lfcins [11, 12]. Moreover, bLfcin had post-antibiotic effect against *S. aureus* and *E. coli* [13]. Therefore, therapeutic molecules can be designed and developed based on the molecular structure of bLfcin, with the purpose of anti-infection caused by pathogenic bacteria [10, 14, 15].

*T. pyogenes* is an opportunistic pathogen that is part of the biota of skin and mucous membranes of upper respiratory, gastrointestinal, urogenital, and mammary tracts of mammals [16]. *T. pyogenes* causes purulent inflammatory responses, such as metritis, mastitis, pneumonia, and abscesses, which generate significant economic losses in animal production. High ratio of *T. pyogenes* isolates from bovine mammary with mastitis suggests that this pathogen play an important role in mastitis [17]. Because of extensive parenchyma destruction and low recovery rate of the mammary quarters, cows with mastitis caused by *T. pyogenes* often tend to be culled. So, *T. pyogenes* takes its immense toll to the dairy industry [18]. At present, many antimicrobial agents, such as beta-lactams, tetracyclines, and macrolides, are used to treat diseases caused by *T. pyogenes* infections [19, 20]. However, the treatment efficacy of these antimicrobial agents is not satisfactory as the antimicrobial resistance in *T. pyogenes* becomes an emerging problem in dairy industry [16, 21, 22].

Previous studies indicated that the cyclic peptides produced by a bridge of disulfide bond tend to change structures and antimicrobial activity of some peptides [23–25]. However, there is little information evaluating changes of molecular structures and antibacterial activities of bLfcin caused by effect of disulfide bond. In the present study, bLfcin and its derivatives, bLfcin with a disulfate bond (bLfcin DB) and bLfcin with a mutation (bLfcin C36G), were synthesized. The secondary structures of these peptides were analysed using circular dichroism (CD) spectra in four solutions with different ionic and hydrophobic strength. Antibacterial activity of these peptides against *T. pyogenes*, isolated from mastitis milk were determined. The results of the present study will be beneficial to the development of new preventive or therapeutic agents for bovine mastitis.

## Results

### Peptide synthesis

**Fig. 1:**
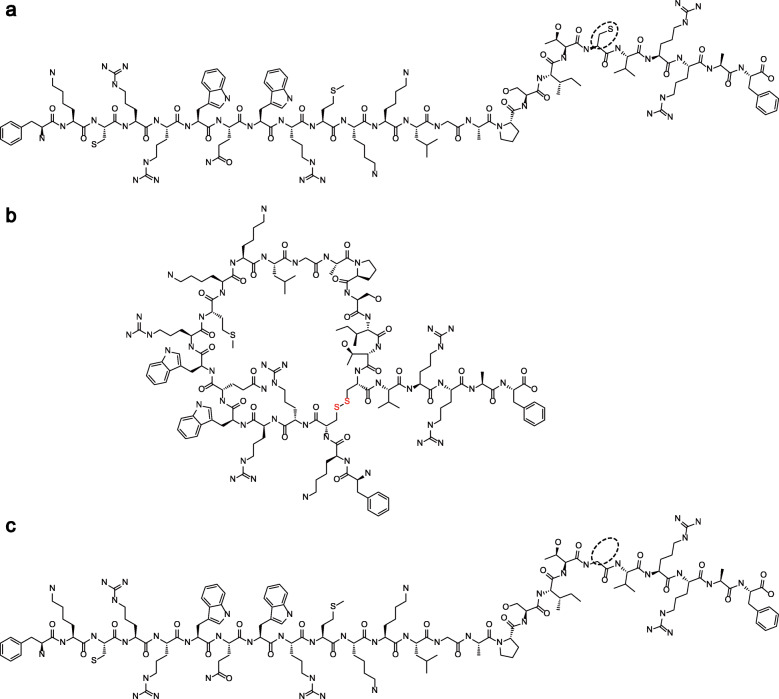
Molecular structures for bLfcin and its derivatives. The molecular structures of (**a**) bLfcin, (**b**) bLfcin DB, and (**c**) bLfcin C36G are shown here. The dashed ellipses show the difference between the mercaptomethyl of bLfcin and the hydrogen base of bLfcin C36G

The peptides were designed and synthesized successfully using solid-phase peptide synthesis (SPPS), specifically: peptides bLfcin, bLfcin DB, and bLfcin C36G. The crude synthetic products were purified by solid-phase extraction chromatography. For all synthesized peptides, the chromatographic profiles determined by reverse phase-high performance liquid chromatography (RP-HPLC) exhibited a purity of more than 95%. The peptides detected by matrix assisted laser desorption/ionization-time of flight-mass spectrometry showed the expected molecular weights. The molecular structures of the synthesized peptides are displayed in Fig. 1. Molecular weight and purity of these peptides are provided in Table 1.

**Table 1:** Synthetic peptides tested for antibacterial activity

Peptides	Amino acid sequences (N-terminal to C-terminal)	Length (AA)	Molecular weight	Purity(%)
bLfcin	FKCRRWQWRMKKLGAPSITCVRRAF	25	3125.82	> 95
bLfcin DB	FKCRRWQWRMKKLGAPSITCVRRAF (with a disulfide bond)^a^	25	3123.82	> 95
bLfcin C36G	FKCRRWQWRMKKLGAPSITGVRRAF^b^	25	3079.73	> 95

^a^The cysteines forming a disulfide bond are shadowed; ^b^the glycine mutation from the cysteine is boxed

### CD spectra and secondary structures

**Fig. 2:**
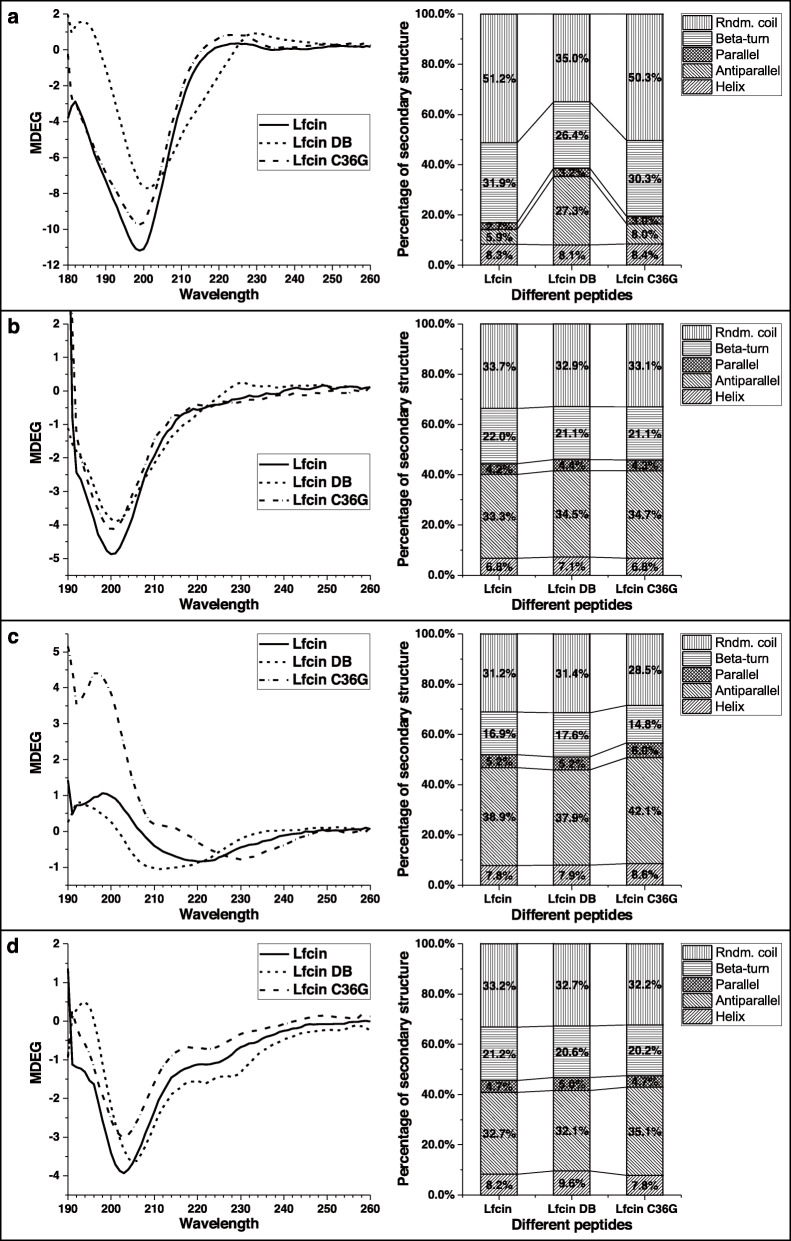
CD spectra curves and secondary structure percentages of bLfcin and its derivatives. The peptides were detected in H_2_O (**a**), PBS (**b**), PBS with 0.56 mM SDS (**c**), and PBS with 8.33 mM SDS (**d**) solution. The CD spectra curves and the secondary structure percentages are indicated on the left and right, respectively

The curves of CD spectra and secondary structures for the synthesized peptides are shown in Fig. 2. (1) The curves show that bLfcin DB had more antiparallel beta-sheets and less random coils and beta-turns compared with bLfcin and bLfcin C36G, and similar ratios of secondary structures of bLfcin and bLfcin C36G were found in H_2_O (Fig. 2a). The percentages of the random coils in bLfcin and bLfcin C36G reached more than 50% in H_2_O, indicating that the two peptides existed mainly in the form of irregular structures, while bLfcin DB kept in ordered molecular structure in comparison to the other peptides (Fig. 2a). (2) In PBS, the three peptides exhibited similar waveforms of the curves. The five types of secondary structures had similar ratios in the three peptides (Fig. 2b). (3) In PBS with 0.56 mM SDS, bLfcin and bLfcin DB possessed the similar waveforms of the curves and the approximate percentages of secondary structures. However, bLfcin C36G had more antiparallel, and less parallel and random coils, compared with the two peptides (Fig. 2c). (4) In PBS with 8.33 mM SDS, the three peptides exhibited similar waveforms of the curves. The five types of secondary structures had similar percentages in the three peptides (Fig. 2d). Each peptide had nearly equal proportions of the secondary structures, compared with those in PBS solution.

### Inhibition halo

In the susceptibility assays, the three antibacterial peptides demonstrated antibacterial activity against *T. pyogenes* isolates, *T. pyogenes* ATCC 19,411 and *E. coli* ATCC 25,922. The antibacterial activities of the peptides were stronger against the two strains of *T. pyogenes* than *E. coli* ATCC 25,922. Among the three peptides, bLfcin DB exhibited the greatest inhibition halos of the *T. pyogenes*.

### Minimum inhibitory concentration and minimum bactericidal concentration

In line with the agar disk diffusion (ADD) experiments, all the three synthetic peptides exhibited more potent antibacterial activity against *T. pyogenes* (MIC_90_ from 3.9 to 15.6 µg/mL) than *E. coli* ATCC 25,922 (MIC_90_ from 62.5 to 250.0 µg/mL) (Table 2). The three peptides demonstrated similar antibacterial activity against the *T. pyogenes* strains derived from the different sources. The peptide bLfcin DB exhibited greater antibacterial activity compared with bLfcin and bLfcin C36G against the *T. pyogenes* strains from the two sources. bLfcin DB demonstrated a comparable antibacterial activity against the *T. pyogenes* isolates and the *T. pyogenes* ATCC 19,411, with the same MIC_50_, MIC_90_, and MBC of 3.9 µg/mL (1.2 µM) (Table 2).

**Table 2:** Antibacterial activity of the designed synthetic peptides against *T. pyogenes* and *E. coli*

Strain	Antibacterial index	Lfcin	Lfcin DB	Lfcin C36G
*Trueperella pyogenes* isolate	MIC_50_	3.9 (1.2)	3.9 (1.2)	7.8 (2.5)
	MIC_90_	7.8 (2.5)	3.9 (1.2)	15.6 (5.0)
	MBC	7.8 (2.5)	3.9 (1.2)	15.6 (5.0)
*Trueperella pyogenes* ATCC 19,411	MIC_50_	3.9 (1.2)	3.9 (1.2)	7.8 (2.5)
	MIC_90_	3.9 (2.5)	3.9 (1.2)	7.8 (5.0)
	MBC	7.8 (2.5)	3.9 (1.2)	15.6 (5.0)
*Escherichia coli* ATCC 25,922	MIC_50_	62.5 (20.0)	125.0 (40.0)	125.0 (40.6)
	MIC_90_	62.5 (20.0)	250.0 (80.0)	125.0 (40.6)
	MBC	62.5 (20.0)	250.0 (80.0)	125.0 (40.6)

Note: The MIC_50_ and MIC_90_ represent the concentrations required to inhibit 50 and 90% of the strains, respectively; MIC_50_, MIC_90_, and MBC in µg/mL (µM)

### Killing kinetic assays

**Fig. 3:**
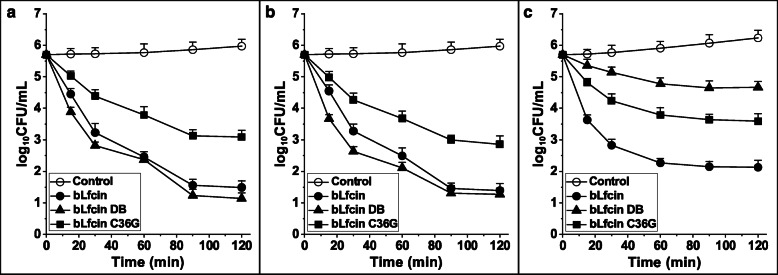
Time-kill curve of the peptides. *T. pyogenes isolates* (**a**), *T. pyogenes* ATCC 19411 (**b**), and *E. coli* ATCC 25922 (**c**) were co-incubated with bLfcin, bLfcin DB, and bLfcin C36G respectively at MBC of bLfcin (7.8 μg/mL for *T.pyogenes*, 62.5 μg/mL for *E.coli*). Y-axis represents the logarithm of CFU. X-axis represents the time point after incubation with peptides. The control is given by bacteria without peptide. Data point are shown by the mean ± standard deviation derived from three repeated experiments

In each instance, the rate of *T. pyogenes* killing by the peptides was time dependent, as well as *E. coli* (Fig. 3). Both bLfcin and bLfcin DB were found to cause 99.9% killing of *T. pyogenes* isolates and *T. pyogenes* ATCC 19,411 within 60 min when used at MBC of bLfcin (7.8 µg/mL) (Fig. 3a,b). However, only bLfcin caused 99.9% killing of *E. coli* cells at MBC of bLfcin (62.5 µg/mL) in 60 min (Fig. 3c). By comparing the killing kinetics of the peptides at the same molar concentration of 7.8 µg/mL (corresponding to MBC for bLfcin, 2 × MBC for bLfcin DB, and MBC/2 for bLfcin), the activity of bLfcin and bLfcin DB toward the two sources of *T. pyogenes* resulted to be much higher than that of bLfcin C36G. Compared with bLfcin, bLfcin DB displayed rapid killing within 60 min, which still caused a reduction in cell viability. A much faster killing rate was demonstrated by bLfcin DB within 15 min (Fig. 3a,b).

## Discussion

Many studies focused on the mechanism towards determining how a peptide would exhibit antimicrobial activity; but a distinct and predictive mechanism was not identified. However, it is a consensus that most AMPs are composed of cationic amino acids to promote selective binding to anionic surfaces of bacteria and hydrophobic amino acids to facilitate partitioning into bacterial membranes [26]. bLfcin has eight basic amino acids, including five arginines and three lysines. These are the basic amino acids implicated in assembly and structure of membrane proteins, which are strongly partitioned into anionic vesicles [27]. The increasing ratio of basic amino acids in potent antibacterial peptides can enhance the effectiveness against certain bacteria pronouncedly [28]. On the other hand, bLfcin has 10 hydrophobic residues in each peptide, some of them with high hydrophobic parameters, such as Ile, Val, and Leu. These hydrophobic residues may induce membrane disturbance by interacting with lipid bilayer, leading to membrane instability, permeability, and even rupture [12, 28–32]. Furthermore, nearly all the hydrophobic residues of bLfcin line up on one face of the peptide and most of the basic groups on the opposite face. The hydrophobic residues and basic residues can interact with each other to influence their functions. For example, the hydrophobic content of peptides determines whether a hydrophobic residue substitution of an interfacial basic residue increases or decreases membrane permeation and bactericidal activity [33]. In addition, aromatic amino acids are vital in the antibacterial function of bLfcin. Substitution of Trp6 or Trp8 in bLfcin by Ala eliminates the antimicrobial activity [34]. Aromatic sidechains can also interact with positively charged groups to form energetically favourable cation-π interaction [34]. The statistical analysis showed that cation-π interactions occurred frequently in high-resolution protein structures, particularly involving Trp and Arg residues [35]. The hydrophobicity, aromaticity, planarity, and amphipathic properties of the indole ring and its ability to form hydrogen bonds could all contribute to its membrane-binding properties and antimicrobial activity [36, 37].

In the present study, Gly was selected as a substitution of Cys, because it is a neutral amino acid, which can reduce the interference of the amino acid itself to the peptide structure, and substituting Cys36 of Lfcin with Gly can prevent forming disulfide bond. The results of the secondary structure globally indicated that the peptides were inclined to exhibit the structural features of a random coil in H_2_O than in the other solutions. However, the PBS solution could augment well-organized structures of the peptides and reduced the ratios of random coils, suggesting that saline ions were conductive to the correct folding of the peptides. The β-turns of the three peptides had the highest percentages in the H_2_O among the four solutions. This was in accordance with a previous study showing that the β-turn for Lfcin17-30 was much better defined in a buffer with lower ionic strength compared with a buffer with higher ionic strength [38]. Meanwhile, a previous study demonstrated that the observed higher bactericidal activity seemed to correlate well with an increase in secondary structures at low ionic strength [38]. These results suggested that bLfcin should be applied as a antibacterial agent in solution with an appropriate ionic strength.

Critical micelle concentration (CMC) is the minimum concentration at which surfactant molecules are associated to form micelles as a model of biological membrane in an aqueous solvent [39]. SDS at CMC (8 mM) in an aqueous solution can form membrane-mimetic SDS micelles, which comprise one inner core formed by aggregation of hydrophobic groups and one outward shell generated by hydrophilic bases in contact with H_2_O. The outward shell possessing negatively charged sulfonic acid groups is similar to negatively charged bacterial membranes. This study showed that the three peptides exhibited similar waveforms of the curves in PBS or PBS with 8.33 mM SDS (a little higher than CMC) solution, and each peptide had alike waveforms in each solution, implying that the model of ordinary biological membrane did not influence the molecular structures of the tested peptides, and the disulfide bond did not affect the structures of Lfcin in these solutions yet.

The conventional micellar carriers disassemble into free surfactants when diluted at concentrations below the CMC [40]. In this study, the solution with lower SDS concentration (0.56 mM) can change secondary structures ratios of the peptides, with the scale of antiparallel going up and the scale of beta-turn descending. bLfcin and bLfcin DB had similar ratios of the secondary structures. Compared with bLfcin and bLfcin DB, bLfcin C36G had more antiparallel and fewer beta-turns at the lower SDS concentration, suggesting that the mutation C36G of bLfcin unable to form a cyclic conformation by a disulfide bond affected the secondary structural elements of the motif.

In the four solutions, bLfcin DB had the least variation range among the three peptides, implying that the disulfide bond could confine the peptide to a finite area, resulting in the secondary structure of bLfcin DB not being able to change relatively freely in varying ionic strength, hydrophobic effect, and membrane environment. Especially in H_2_O, bLfcin DB had more well-organized structures compared with the others. Furthermore, the disulfide bond could improve antibacterial functional execution of bLfcin DB through stabilizing its molecular structure by the disulfide bond. A much faster killing rate was displayed by bLfcin DB in killing kinetic assays, compare with the other peptides, suggesting that bLfcin DB targeted bacterial membrane to cause bacterial growth inhibition. These phenomenon implied that the disulfide bond was crucial for the preservation of the molecular structure of bLfcin, and beneficial to executing antibacterial function.

Although the effects of membrane-mimetic SDS micelle and hydrophobicity of SDS on the secondary structures of the tested peptides were analysed, the real interaction between the peptides and the bacterial membranes could not be completely simulated because the real bacterial membrane compositions are more complicated than those of the model membranes. With current technologies, the state of bLfcin and its derivatives cannot be tested by CD spectra due to the complicated conditions of bacterial membrane surface and cultural components. Furthermore, whether the disulfide bond is formed within the molecule of bLfcin on the membrane surface of bacteria is still not clear based on the results of this study. However, this study confirmed that bLfcin and its two derivatives could transform their structure under alterative ionic strengths and hydrophobic effects, and the forming of disulfide bond was beneficial to its antibacterial activity against *T. pyogenes*.

## Conclusions

In summary, this study showed that the synthetic peptide of bLfcin DB exhibited a high degree of antibacterial activity against *T. pyogenes*, even higher than that of bLfcin and bLfcin C36G. The finding suggested that the formation of disulfide bond could be helpful to execute higher antibacterial activity against these bacteria and could be considered for the development of new prophylactic or therapeutic agents against diseases mainly caused by *T. pyogenes*. The results of the present study indicated the value of SPPS in designing and obtaining new AMP-based therapeutic agents.

## Methods

### Peptide synthesis

The peptides were synthesized by manual SPPS using the manual Fmoc/tBu strategy. Briefly, Rink amide resin (AAPPTec, KY, USA) (0.46 meq/g) was used as a solid support. (a) The Fmoc group was removed by treating with 20% 4-methylpiperidine (Sigma-Aldrich, MO, USA) in N,N-dimethylformamide (AAPPTec, KY, USA). (b) For the coupling reaction, N,N-dicyclohexylcarbodiimide (AAPPTec, KY, USA)/ 6-chloro-1-hydroxy-benzotriazole (AAPPTec, KY, USA) (0.20/0.21 mmol) was applied to preactivate Fmoc-amino acids (AAPPTec, KY, USA) (0.21 mmol) in N,N-dimethylformamide at room temperature. (c) A cleavage cocktail containing trifluoroacetic acid (TFA)/H_2_O/triisopropylsilane (Sigma-Aldrich, MO, USA)/1,2-ethanedithiol (Sigma-Aldrich, MO, USA) (93/2/2.5/2.5 v/v/v) was used for side chain deprotection reactions and peptide separation from the resin. (d) Cool diethyl ether (Sigma-Aldrich, MO, USA) was used for precipitation of crude peptides, which was dried at room temperature and analysed using RP-HPLC analytical chromatography.

### Peptide purification and identification

The crude peptides were purified by solid-phase extraction chromatography. A Supelclean LC-18 SPE column was used for the purification process. The crude peptides were passed through the column, and the elution was implemented using a gradient of solvent B (0.1% TFA in ACN (Sigma-Aldrich, MO, USA)). The collected fractions were analysed by RP-HPLC and MS. For RP-HPLC analysis, 20 µL of crude peptide stock solution (1 mg/mL) was analysed on a C18 column (Kromasil; 5 µm; 4.6 × 150 mm) using an Agilent 1200 liquid chromatograph (NE, USA). A linear gradient was employed, from 20–50% solvent B in solvent A (0.1% TFA in H_2_O). The gradient time was 25 min. A wavelength of 220 nm and a flow rate of 1.0 mL/min for detection were used at room temperature. Matrix assisted laser desorption/ionization-time of flight-mass spectrometry analysis was performed on an Ultraflex III TOF-TOF mass spectrometer (Bruker Daltonics, Bremen, Germany) in the reflectron mode, using an MTP384 polished steel target (Bruker Daltonics), 2,5-dihydroxybenzoic acid, or sinapinic acid as a matrix, 500 shots with a 25–30% power laser.

### CD analysis

CD spectra of samples were acquired on a spectropolarimeter (Applied Photophysics Ltd, UK) [41]. Samples containing 0.16 mg/mL purified synthetic peptide dissolved in H_2_O, PBS (5.8 mmol/L phosphate, 58.3 mmol/L NaCl, pH 7.0), PBS containing 0.56 mmol/L SDS, or PBS containing 8.33 mmol/L SDS were recorded at room temperature in a 0.5-mm-pathlength quartz cuvette. The bandwidth was set to 1.0 nm. Far-UV spectra between 180/190 nm and 260 nm were collected using a 1.0 nm step size. The response time was set to 0.5 s. The results of three spectra were accumulated and then averaged to generate mean spectra. A blank spectrum without peptides was recorded for each buffer and subtracted in the final analysis. Mean residue ellipticity [θ] was calculated using the following formula:

$$MRE=\left(\theta\times{0.1}\times{MRW}\right)/cl$$

where *θ* is the measured ellipticity in mdeg, *c* is the concentration in mg/mL, *l* is the cuvette pathlength in cm, and *MRW* is the mean residue weight of the peptide. *MRW* is defined as

$$MRW=MW/\left(n-1\right)$$

where *MW* is the molecular weight of the peptide in daltons, and *n* is the number of residues in the peptide.

### Bacterial isolation

To evaluate the effect of the disulfide bond on the antibacterial activity of bLfcin, *T. pyogenes* was separated from cow milk with mastitis. Mastitis milk samples of cows were obtained from commercial dairy herds located at Gansu province in China. After delivered to laboratory, 10 µl of milk sample was inoculated onto a trypticase soy agar (TSA) plate containing 5% fetal bovine serum or a MacConkey agar (Oxoid, United Kingdom) and cultivated at 37 °C for 36 to 48 h. Colonies with the dark pink to red colours were further confirmed by the Vitek system (bioMérieux, France). A total of 5 *T. pyogenes* isolates were identified as described previously [42], and these isolates belonged to the same strain. The isolated strains were grown on TSA plates containing 5% fetal bovine serum and cultured at 37 °C under 5% CO_2_.

### Susceptibility assays

The susceptibility assays of three *T. pyogenes* isolates were carried out by ADD. The reference strains *T. pyogenes* ATCC 19,411 and *E. coli* ATCC 25,922 were used as controls. Briefly, an inoculum aliquot (200 µL; 2 × 10^6^ colony forming units (CFU)/mL) was placed on Mueller-Hinton agar (MHA) (ATCC, VA, USA). The ADD assays were carried out at only a one concentration for each peptide (400 µg/disc), followed by the incubation for 48 h at 37 °C. As a positive control, 1.25 µg/mL ciprofloxacin was used for all tested strains. Sterile water was used as a growth control. Each of these tests was performed three times.

### Antibacterial activity assays

Because there was no CLSI method available for the determination of MICs for *T. pyogenes*, and there were no published MIC breakpoints for AMP in the CLSI criteria or the literature, the susceptibility assays of the isolated strains were carried out by broth dilution methods that have been reported previously [43]. The strains *T. pyogenes* ATCC 19,411 and *E. coli* ATCC 25,922 were selected as control groups. The MIC and MBC were determined using the following microdilution assay. In brief, the bacterial strains were incubated at 200 rpm for 12 to 15 h, at 37 °C, in a Mueller Hinton broth (MHB) (ATCC, VA, USA) until an optical density of 0.15 to 0.30 (620 nm) was obtained. Then, 75 µL of 2 × AABSA solution (0.02 µL/mL acetic acid and 0.4 mg/mL BSA) containing peptide was added to each well on the first column of a 96-well microtiter plate. The peptide serial dilution was performed by the 2 × AABSA solution from the first column to the tenth column. Then, the peptide solution was mixed with 75 µL of the inoculum (1 × 10^6^ CFU/mL) in each cell. The concentrations of peptide serial dilution were halved, and the final serial concentrations were 1000, 500, 250, 125, 62.5, 31.25, 15.63, 7.81, 3.91, and 1.95 µg/mL, respectively. At the same time, the concentration of the bacteria reached 5 × 10^5^ CFU/mL, in line with previous finding [43]. The solutions were incubated at 200 rpm at 37 °C for 28 h, and the absorbance at 620 nm was measured using an Asys Expert Plus ELISA reader. To determine the MBC, an aliquot was taken from each well and was spread onto a MHA plate. After 24 h at 37 °C, the CFU/mL was determined. All the tests were performed in duplicate and repeated three times.

### Killing kinetic assays

The test strains were cultured to reach exponential growth phase at 37 °C in MHB. The strains were then adjusted to achieve cell density of 1.0 × 10^6^ CFU/mL in MHB using a nephelometer. The peptide solutions at 2-fold MBC of bLfcin were added to isopyknic bacterial medium in culture tubes. Bacteria reached a final cell density of 5.0 × 10^5^ CFU/mL, and the peptide concentrations reached MBC of bLfcin. The bacteria and peptide mixtures were incubated for time periods up to 120 min at 37 °C. Aliquots of cultures were taken at 0, 15, 30, 60, 90, and 120 min post-inoculation. Serial 10-fold dilutions of each aliquot was prepared in basal medium and plated in triplicate onto suitable agar media. Viable bacterial counts were enumerated by a standard dilution and plating method. Time-kill curves were constructed by plotting log_10_CFU/mL against the time of incubation. Killing assays were performed in triplicates and the results were averaged.

## Data Availability

All the data used to support the findings of this study is available in the manuscript. Raw datasets may also be requested from the corresponding author provided that all ethical requirements have been met.

## Abbreviations

*T. pyogenes*: *Trueperella pyogenes*
*E. coli*: *Escherichia coli*
*S. aureus*: *Staphylococcus aureus*
AMP: Antimicrobial peptide
Lfcin: Lactoferricin
bLfcin: Bovine Lfcin
bLfcin DB: bLfcin with a disulfate bond
bLfcin C36G: bLfcin with a mutation
CD: Circular dichroism
SPPS: Solid-phase peptide synthesis
RP-HPLC: Reverse phase-high performance liquid chromatography
ADD: Agar disk diffusion
CMC: Critical micelle concentration
TFA: Trifluoroacetic acid
TSA: Trypticase soy agar
MHA: Mueller-Hinton agar
MHB: Mueller-Hinton broth
CFU: Colony forming units
MIC: Minimum inhibitory concentration
MBC: Minimum bactericidal concentration
